# Dentition: A Reply to Medical Criticism

**Published:** 1892-12

**Authors:** 


### Editorial.

### DENTITION: A REPLY TO MEDICAL CRITICISM.

This subject seems to have started anew with our brethren of the medical profession, judging by the two articles which we publish in this number, one original and the other under the head of "Selections."

It is passing strange to the dental mind that the medical profession should at this late day be gravely discussing the question whether "dentition produces general or reflex symptoms." It seems to prove that, with all the intelligence manifested in other directions, the relations the teeth maintain to the rest of the organism has yet to be comprehended.

While it is true that all diseases of infancy, and we may say childhood, cannot be ascribed to dentition, there are certain well-marked conditions, unmistakable to the trained intelligence, produced by reflex disturbance, and we fail to understand why these are not recognized.

It is difficult to believe that our medical friends are not familiar with the anatomical features surrounding the developing tooth and fail to recognize the possibilities connected therewith, yet a careful reading of the two articles named leads directly to such a conclusion.

It certainly must be conceded that teeth are not developed at once, but by slow and very gradual processes. If these be continued with an harmonious growth of contiguous and related parts, dentition may proceed in the deciduous series with but slight reflex disturbance; but, if this be not the case, growth means pressure on the nerves supplying the pulp, followed by irritation to be felt possibly throughout the organism, producing new centres of irritation and new foci of inflammation.

A very remarkable statement is that of the editor of the *Journal of the American Medical Association*, that "we think it can be said, without fear of contradiction, that there is not a single positive observation which has ever been recorded to prove that dentition produces general or reflex symptoms. It is undeniable that at the period in life when dentition is in progress the infant is subject to certain disorders which occur much more commonly than at any other period of life."

Again, Dr. Patterson reiterates this idea in his paper when he quotes from the editor of the *Dental Cosmos*: "What proof have we that otalgia, otorrhœa, deafness, amaurosis, hemicrania, neuralgia, hysteria, chorea, epilepsy, tetanus are produced by dentition?" And then adds, "The hurricane of progressive medicine has swept away such wild and speculative theories."

It would seem a waste of energy to quote to men who must be already familiar with the literature of the subject, but if they wish to know of cases of reflex disturbance minutely described, we can refer them to Dr. Georg Creniceanu (appendix to "Diagnostik der Zahnkrankheiten," Arkövy), in his valuable paper on diseases of the eye produced by pathological conditions of the teeth. These are given in detail, and should at least cause serious reflection.

Coming nearer home, and more readily procurable, they will find the entire subject considered with marked ability by Dr. Albert P. Brubaker, in his exhaustive paper in the third volume of the "American System of Dentistry." We would ask their attention to the following quotation: "It is a reasonable supposition that the laws of nerve-force correspond to the same general laws as other forces. As no force is ever lost, but must, when it disappears, appear as some other mode of motion, so peripheral irritation—continually travelling inward—is either reflected over and outward by other routes to other organs, or is stored up in interrelated centres until morbid function results in them."

It may be objected that the statements made are mainly confined to diseases of the permanent teeth, but the law of force applicable in the one case must be general in its operation, or it ceases to be a law. We have not the space to quote in full from authorities on this subject, nor is it necessary; but the following from the pen of Dr. James W. White—in the "American System of Dentistry"—clearly expresses the idea that greater possibilities of reflex disturbance are usual in infancy than in maturer years, a conclusion borne out by the observation of the writer: "Owing to the predominance of the spinal system in infancy, this sympathy of distant organs with one another is notably greater than in adult life, creating a special tendency to the production of reflex phenomena. The exceeding mobility of the nervous system at this period is also such that a mere peripheral irritation is liable to result in sympathetic general disturbance,—often, indeed, to the overshadowing of the original lesion by accessory phenomena."

It is certainly too late in the progress of medical science to assert that no reflex disturbance occurs in teething, or that it is solely dependent upon the eruption through the gum-tissue. Such a statement seems to indicate a blind perversion of facts, and which could never be made truthfully by one who had watched the progressive phenomena resulting from dentition and the relief afforded by the use of the lance. Condie ("Diseases of Children") relates the following case, which, while an extreme one, could be duplicated in degree in the experience of many: "A child, after having suffered greatly from difficult dentition, apparently died and was laid out for interment. M. Lemonnier, having some business at the house of the nurse with whom the child resided, after fulfilling the object of the visit was desirous of ascertaining the condition of the alveola. He accordingly made a free incision through the gums, but on preparing to pursue further his examination he perceived the child to open its eyes and give other indications of life. He immediately called for assistance, the shroud was removed from the body, and by carefnl and persevering attention the child's life was saved. The teeth in due time made their appearance."

No consideration of dentition would be complete that failed to estimate the possibilities of reflex disturbance in the eruption of the permanent series, together with the reasons that cause special phenomena. The writer of "The Dental Aspect of It," in this number, may have reason for his opinions in experiences with teeth of English patients, not attainable in this country with our mixed population. The type of teeth may be nearly or quite established in Great Britain, which gives the greatest harmony of development and the least liability to reflex irritation. That perfection has not been reached on this continent, and the death-rate will continue to extend indefinitely until that desirable end is accomplished.

The teeth anterior to the second permanent molar rarely give recognizable systemic disturbance as they develop gumward. The first permanent molar being developed in the body of the inferior maxilla, where ample room ordinarily exists, is erupted without special notice, but this happy condition of affairs cannot as a rule take place with the second molar in the same jaw. We speak of the inferior maxilla, as it is here that in our opinion most, if not all, the reflex phenomena originate.

Why should the second permanent molar produce unusual irritation and be a source of disturbance to a greater extent than the first? The answer will be found in the fact that these teeth are developed in the rami of the jaw, and in assuming the direct vertical position the space at the angle is frequently not sufficient to allow for their development. The process of formation of the dental tissues proceeds regardless of this, and the result is pressure upon the inferior dental nerve and possible cerebral disturbance to an alarming extent. As this stage antedates by several years the proper time for eruption, the question of the gum having a part in the lesion need not be considered. The writer of this has had occasion to relieve convulsions, unquestionably arising from this tooth, at least four years prior to its final eruption. The proof that it originated here was furnished by the fact that lancing would relieve the seizure, and when the case was carefully watched in anticipation, lancing would invariably prevent it. If our correspondent will carefully examine a child's skull about the age of nine years, he will, we think, come to the conclusion that the possibilities of irritation are not remote.

The reflex disturbances of the third molar are so well known that it seems useless to more than mention them. A case of a misplaced wisdom tooth which came under the care of the writer was the cause of not only untold suffering through eighteen years, but resulted in great loss of vision, and deafness. Both the eye and ear assumed nearly their normal functions on the removal of the cause. It must have come to the notice of every aurist, as it certainly has to every dentist, that the so-called earache of childhood is often the result of an exposed pulp, and the relief given the latter cures the former. The neuroses dependent on various pathological conditions of the teeth are so well understood that no further mention need be made of them.

The perforation of the gum has been regarded by almost all medical writers as the primal cause of all the phenomena attendant upon dentition. That this is an error is clearly manifest to every observant dentist. The gum is by no means a very sensitive portion of the organism, and, were it not for the causes enumerated, there would be but little trouble attending the process. The part the gum takes in this is to hold down the entire body of the developing tooth, producing the pressure before mentioned, the exception being the second molar. If resorption of this tissue proceeds in proportion to development, there is no untoward result, but if not, then means must be resorted to to relieve the tension. To wait until this is manifested by the upward extension of the gum is simply to fail to understand conditions, for, as Dewees says ("Treatise on the Physical and Medical Treatment of Children"), "Though the tooth cut upon may be yet remote from the surface, the operation (lancing) may be of the greatest possible advantage."

The statement of Dr. Patterson that "this pathological condition (inflammation) has its origin in micro-organisms has yet to be proven; and that they have anything to do with the systemic disturbance of dentition, except as a secondary factor in the increase of inflammation in near or remote parts, is not established. The whole theory of inflammation remains to-day very much as it was left ten years ago, notwithstanding the advances made in bacteriological research. The theory that bacteria are the cause of lesions in dentition has no support to sustain it, unless the phagedenic condition of the gums covering the erupting teeth be regarded as an illustration. This, however, is a very exceptional condition, resulting from causes existing in the oral cavity, and entirely independent of dentition as we understand it. Where ulceration is present it means simply local inflammation without systemic disturbance, unless it extends to the pericementum, as is frequently the case in the wisdom teeth.

The assertion that micro-organisms can "affect the tissue in question through the circulation" is not fully supported; indeed, is at variance with the investigations of some of the best minds in the medical profession. Attention is called to the report of M. Peyrot, at the Fifth Surgical Congress, in Paris, 1891; also to the sterility of pus in abscess of the liver, by Kartoulis and Lavaran; to the investigations of Janowski in regard to creolin; to the results obtained by G. Lemaire in injections of mercury, and finally to the conclusions arrived at by Kreibohm, Rosenbach, Grawitz, and de Bary, quoted by Miller. The question still remains an open one, and dogmatic assertions are, as yet, out of place.

We would recommend our medical friends to make a thorough re-examination of this important subject, satisfied that if this be done new light will dawn upon many pathological problems as yet obscure to their minds.

